# In-vitro evaluation of fracture resistance of teeth restored with different high-viscosity glass ionomer restorative materials and bulk-fill composite resins

**DOI:** 10.1007/s00784-024-05745-9

**Published:** 2024-05-29

**Authors:** Merve Nezir, Suat Ozcan

**Affiliations:** Department of Restorative Dentistry, Faculty of Dentistry, Gazi University, Bişkek St. 1.St. Number:8, Çankaya, Ankara, 06490 Turkey

**Keywords:** Bulk-fill, Closed-sandwich technique, Fracture resistance, Glass ionomer, Open-sandwich technique

## Abstract

**Objectives:**

This study aimed to evaluate the effect of restorations made with a glass-hybrid restorative system (GHRS), a high-viscosity glass ionomer restorative material (HVGIC), a high-viscosity bulk-fill composite resin (HVB), a flowable bulk-fill composite resin (FB), and a nanohybrid composite resin (NH), which are commonly preferred in clinical applications on the fracture resistance of teeth *in-vitro*.

**Materials and methods:**

One hundred intact human premolar teeth were included in the study. The teeth were randomly divided into ten groups (*n* = 10). No treatment was applied to the teeth in Control group. Class II cavities were prepared on the mesial surfaces of the remaining ninety teeth in other groups. For restoration of the teeth, a GHRS, a HVGIC, a HVB, a FB, and a NH were used. Additionally, in four groups, teeth were restored using NH, GHRS, and HVGIC with open and closed-sandwich techniques. After 24 h, fracture resistance testing was performed. One-way ANOVA and Tukey HDS tests were used for statistical analysis of the data.

**Results:**

The fracture resistance values of Control group were statistically significantly higher than those of GHRS, HVGIC, FB, NH, HVGIC-CS, GHRS-OS, and HVGIC-OS groups(*p* < 0.05). There was no statistically significant difference observed between the fracture resistance values of Control, HVB, and GHRS-CS groups (*p* > 0.05).

**Conclusion:**

It can be concluded that the use of HVB and the application of GHRS with a closed-sandwich technique may have a positive effect on the fracture resistance of teeth in the restoration of wide Class II cavities.

**Clinical relevance:**

The use of high-viscosity bulk-fill composite resin and the application of glass-hybrid restorative system with the closed-sandwich technique in the restoration of teeth with wide Class II cavities could increase the fracture resistance of the teeth.

## Introduction

Amalgam, composite resin, and glass ionomer cement (GIC) are routinely used in clinical practice as permanent direct restorative materials [1]. High-viscosity glass ionomer cements (HVGICs) have been developed to enhance the weak mechanical properties of conventional glass ionomer cements (GICs) and increase in resistance to occlusal forces, expanding the indication areas is limited to Class I and V restorations [2].

Another material used as a dental restorative material is bulk-fill composite resins. Nowadays, bulk-fill composite resins are among the preferred materials for direct restorations of teeth. As a result of their increased translucency, they have lower shrinkage rates and higher reactivity than most of the conventional composite resins, thereby increasing the light penetration and polymerization depth [3, 4]. Their ability to be applied in layers of 4–5 mm thickness shortens clinical procedures and makes them easier to use [4]. It has been reported that bulk-fill composite resins exhibit less polymerization shrinkage stress during and after light polymerization in Class II posterior composite resin restorations compared to conventional micro-hybrid composite resins [5].

GICs have various applications, including restorations performed using the sandwich technique. Through the sandwich technique, both the advantages of GICs and composite resins can be utilized. GICs offer benefits such as anti-cariogenic properties, chemical adhesion, fluoride ion release, reduced microleakage, and remineralization potential, while composite resins provide advantages such as bonding to enamel, durability, and aesthetic superiority [6].

One of the methods recommended to eliminate stresses caused by the polymerization shrinkage of composite resin is to use a material with excessive elastic deformation in the early stages of polymerization. Therefore, sandwich restorations, where a part of the composite resin’s volume is replaced with GIC, is one of the techniques proposed to improve marginal adaptation in Class I and II cavities. The sandwich technique is suggested to be used in cavities of medium or larger sizes, typically those that are primarily restored with composite resin [7].

Literature includes studies that evaluate the structural and mechanical properties of different high-viscosity glass ionomer restorative materials, bulk-fill composite resins, and nanohybrid composite resins, as well as studies assessing the changes in the physical and mechanical properties of teeth caused by restorations using these materials [8–15]. However, studies that evaluate these materials together, particularly those assessing the impact of applying high-viscosity glass ionomer restorative material and nanohybrid composite resin with open and closed-sandwich techniques on the fracture resistance of teeth during restoration, are limited.

This study aims to evaluate the impact of restorations made with a glass-hybrid restorative system, a high-viscosity glass ionomer restorative material, a high-viscosity bulk-fill composite resin, a flowable bulk-fill composite resin, and a nanohybrid composite resin on the fracture resistance of teeth *in-vitro*, which have recently become frequently preferred in clinical practice. Additionally, in this study, teeth were restored using a glass-hybrid restorative system, a high-viscosity glass ionomer restorative material, and a nanohybrid composite resin with open and closed-sandwich techniques. The objective is to evaluate the effect of applying these materials with open and closed-sandwich techniques on the fracture resistance of teeth. The first null hypothesis of the study is that the restorative technique used does not affect the fracture resistance of teeth. The second null hypothesis is that there is no difference in the fracture resistance of teeth restored in the control group compared to those restored in the other groups.

## Materials and methods

### Preparation of specimens

In calculating the specimen size, an effect size of 1.63 was considered. To test the statistical significance of differences between any two groups with 95% power and a 5% error level, it was planned to include at least 9 specimens in each group. Considering possible data losses, 10 specimens were planned for each group. Specimen size calculations were performed using the G*Power 3.1.9.6. software package (Franz Faul, University of Kiel, Germany).

The study commenced after obtaining approval from the Gazi University, Faculty of Dentistry Clinical Research Ethics Committee with decision number GÜDHKAEK.2022.04/6 dated 24.02.2022.

The materials used in the study, their contents, and application methods are shown in Table 1.

**Table 1:** Materials used in the study, their contents, and application methods

Material Name	Material Type	Manufacturer	Contents	Application Procedure
Equia Forte HT	Glass-hybrid Restorative System	GC Corp., Tokyo, Japan	Powder: Ultrafine reactive glass particles (fluoroaluminosilicate glass), polyacrylic acid, iron oxide, Liquid: Polybasic carboxylic acid, water	• It was mixed in the amalgamator for 10 s. It was then applied to the tooth surface.
Fuji IX GP	High-Viscosity Glass Ionomer Restorative Material	GC Corp., Tokyo, Japan	Powder: Aluminosilicate glass, polyacrylic acid, Liquid: Polyacrylic acid, water	• It was mixed in the amalgamator for 10 s. It was then applied to the tooth surface.
Tetric N-Ceram Bulk-Fill	High-Viscosity Bulk-Fill Composite Resin	Ivoclar Vivadent, Schaan, Liechtenstein	Filler content: 57% by volume, 81.2% by weight Barium glass, ytterbium trifluoride, mixed oxide, silicon dioxide, prepolymers Bis-GMA, Bis-EMA, UDMA, CQ.	• It was applied in 4 mm layers. • It was polymerized with an LED light device for 10 s.
Estelite Bulk-Fill Flow	Flowable Bulk-Fill Composite Resin	Tokuyama Dental Corp., Tokyo, Japan	Filler content: 56% by volume 70% by weight New organic-inorganic hybrid filler, supra nano spherical filler (silicon dioxide-zirconium oxide), Bis-GMA, TEGDMA, Bis-MPEPP, CQ, Radical-Amplified Photopolymerization initiator	• It was applied in 4 mm layers. • It was polymerized with an LED light device for 10 s.
Gaenial A’chord	Nanohybrid Composite Resin	GC Corp., Tokyo, Japan	Bis-MEPP, filler content: 82 wt% Glass fillers (300 nm barium glass) 16 nm (fumed silica), organic fillers (300 nm barium glass; 16 nm fumed silica)	• It was applied in 2 mm layers. • It was polymerized with an LED light device for 20 s.
Clearfil SE Bond	Universal Bonding Agent	Kuraray Corp., Osaka, Japan	Self-etching/primer: HEMA (20–30), MDP, Hydrophilic aliphatic dimethacrylate, dl-CQ, water, accelerators, coloring agents and others Bond: HEMA (25–35), MDP, Bis-GMA, HEMA, Hydrophobic dimethacrylate, dl-CQ, N,N, diethanol-p-toluidine, Silanated colloidal silica	• Primer was applied for 20 s and gently air dried. • Then, Bond was applied and polymerized with an LED light device for 10 s.
Cavity Conditioner	Cavity Conditioner	GC Corp., Tokyo, Japan	20% Polyalkenoic acid, 3% aluminum chloride (pH 1.2)	• It was applied to the dentin surface for 10 s. • Rinsed with water and dried gently.
Equia Forte Coat	Coating Agent	GC Corp., Tokyo, Japan	Methylmethacrylate, multifunctional methacrylate, CQ	• After it was applied to the material surface, it was polymerized for 20 s.

Abbreviations: Bis-GMA: bisphenol A diglycidylmethacrylate; Bis-EMA: ethoxy bisphenol A-dimethacrylate; UDMA: urethane dimethacrylate; CQ: camphoroquinone; TEGDMA: triethylene glycol dimethacrylate; Bis-MPEPP: 2,2-bis[(4-methacryloxy polyethoxy)phenyl]propane; Bis-MEPP: bis-methacryloxyethyl phenyl propane; HEMA: 2-Hydroxyethyl methacrylate; MDP: 10-Methacryloyloxydecyl dihydrogen phosphate

One hundred intact human premolar teeth with indications for extraction (50 upper premolar teeth and 50 lower premolar teeth) were included in the study. While selecting the teeth, upper premolar and lower premolar teeth with similar sizes were included in the study. Dental plaque, calculus and periodontal tissues of the teeth were removed using a hand scaler and stored in a 0.1% thymol solution for 2 months at room temperature. The premolar teeth were randomly distributed, with 5 upper premolar teeth and 5 lower premolar teeth in each group. Subsequently, the teeth were placed in pink cold-cure acrylic surrounded by a polyvinyl chloride (PVC) cylinder, ensuring that they remained 1.5–2 mm below the cemento-enamel junction and parallel to the occlusal surfaces. The teeth were randomly divided into 10 groups, with 10 teeth in each group (*n* = 10).

**Fig. 1:**
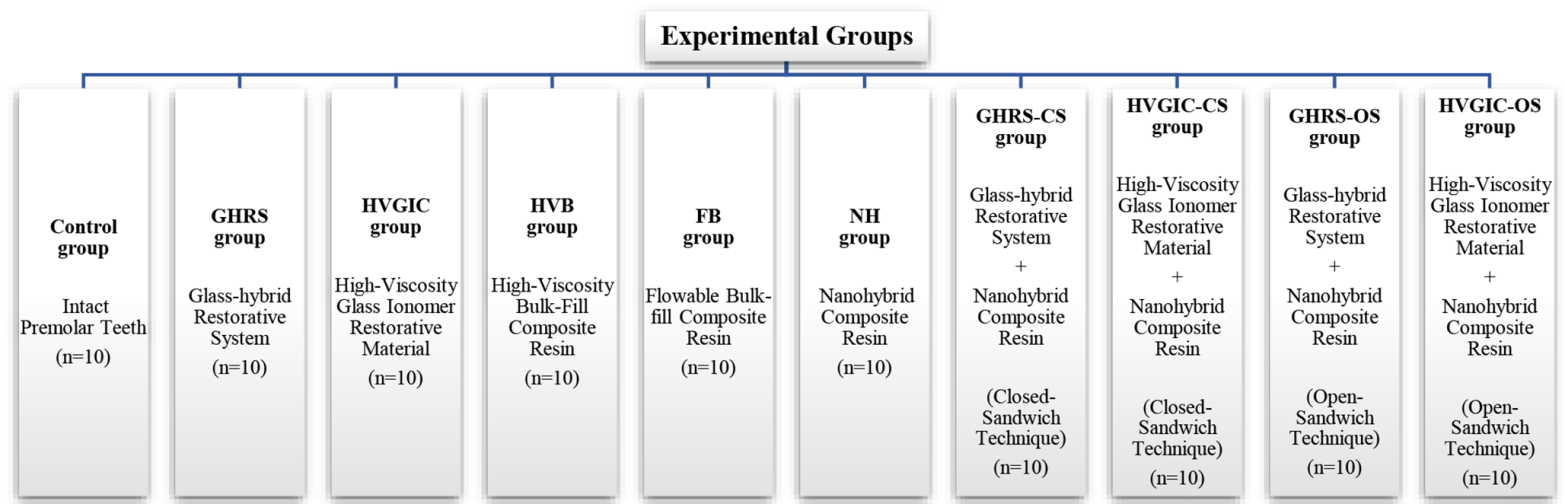
Experimental groups

**Fig. 2:**
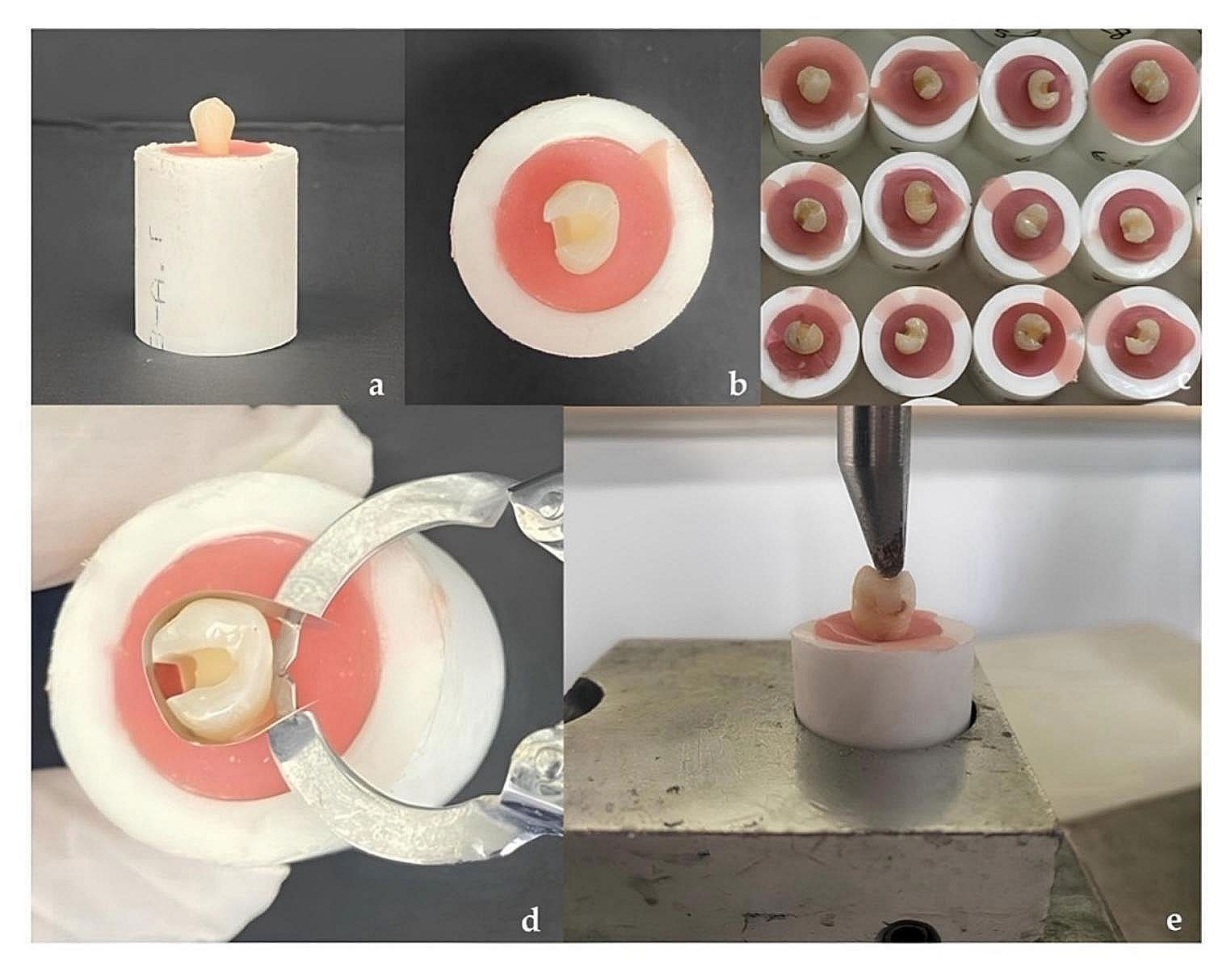
Placing the extracted human premolar tooth in pink cold acrylic (**a**); Preparing of a large Class II mesio-occlusal cavity into the premolar tooth (**b**); cavity-prepared premolar teeth (**c**); placement of ivory matrix band for restoration to the cavity-filled tooth (**d**); Performing the fracture resistance test of the restored premolar tooth on the Universal Testing Device (**e**)

In Control group, no treatment was applied to the teeth. In Groups GHRS, HVGIC, HVB, FB, NH, GHRS-CS, HVGIC-CS, GHRS-OS, and HVGIC-OS, a total of 90 teeth were prepared with wide Class II cavities on their mesial surfaces. The occlusal part of the cavities was prepared to be one-third of the buccolingual width of the tooth and with an occlusal depth of 2.5 mm. The axial wall height was set at 2.5 mm. The proximal step of the cavity was positioned 1.5 mm occlusal from the cemento-enamel junction and prepared to a width of 2 mm. The depth and width of the cavity were measured using a periodontal probe. The diamond fissure bur used was replaced with a new one for every four teeth. After preparation, the teeth were cleaned with compressed air and water. Subsequently, the teeth were restored using an ivory matrix band and an ivory matrix holder (Figs. 1 and 2). The restoration procedure according to the groups was as follows:

- ***Restoration with glass-hybrid restorative system***: After applying cavity conditioner (Cavity Conditioner, GC Dental Corp., Tokyo, Japan) to the exposed dentin surface for 10 s, the glass-hybrid restorative system (Equia Forte, GC Dental Corp., Tokyo, Japan) was mixed for 10 s in an amalgamator and used to restore the teeth. Then, a coating agent (Equia Forte Coat, GC Dental Corp., Tokyo, Japan) was applied to the restoration surface according to the manufacturer’s instructions and polymerized for 20 s using a Light Emitting Diode (LED) curing light device (D-Light Pro, GC Europe N.V., Leuven, Belgium) (High Power [HP] mode, light intensity: 1400 mW/cm^2^).
- ***Restoration with high-viscosity glass ionomer restorative material***: After applying cavity conditioner to the exposed dentin surface for 10 s, high-viscosity glass ionomer restorative material (Fuji IX GP, GC Dental Corp., Tokyo, Japan) was used to restore the teeth. Then, a coating agent was applied to the restoration surface according to the manufacturer’s instructions and polymerized with an LED curing light device.
- ***Restoration with high-viscosity bulk-fill composite resin***: The teeth were restored using a self-etch adhesive procedure. A universal bonding agent (Clearfil SE Bond, Kuraray Corp., Osaka, Japan) was applied to the dentin surfaces according to the manufacturer’s instructions and polymerized with an LED curing light device. Then, high-viscosity bulk-fill composite resin (Tetric N-Ceram Bulk-Fill, Ivoclar Vivadent, Schaan, Liechtenstein) was applied in 4 mm increments and polymerized with an LED curing light device.
- ***Restoration with flowable bulk-fill composite resin***: The teeth were restored using a self-etch adhesive procedure. A universal bonding agent was applied to the dentin surfaces according to the manufacturer’s instructions and polymerized with an LED curing light device. Then, flowable bulk-fill composite resin (Estelite Bulk-Fill Flow, Tokuyama Dental Corp., Tokyo, Japan) was applied in 4 mm increments and polymerized with an LED curing light device.
- ***Restoration with nanohybrid composite resin***: The teeth were restored using a self-etch adhesive procedure. A universal bonding agent was applied to the dentin surfaces according to the manufacturer’s instructions and polymerized with an LED curing light device. Then, nanohybrid composite resin (Gaenial A’chord, GC Dental Corp., Tokyo, Japan) was applied in 2 mm increments and polymerized with an LED curing light device.
- ***Restoration with glass-hybrid restorative system with closed-sandwich technique***: After applying cavity conditioner to the exposed dentin surface for 10 s, a base was performed with glass-hybrid restorative system. Then, a universal bonding agent was applied according to the manufacturer’s instructions and polymerized with an LED curing light device. Subsequently, nanohybrid composite resin was applied in 2 mm increments and polymerized with an LED curing light device.
- ***Restoration with high-viscosity glass ionomer restorative material with closed-sandwich technique***: After applying cavity conditioner to the exposed dentin surface for 10 s, a base was performed with high-viscosity glass ionomer restorative material. Subsequently, a universal bonding agent was applied according to the manufacturer’s instructions and polymerized with an LED curing light device. Then, the nanohybrid composite resin was applied in 2 mm increments and polymerized with an LED curing light device.
- ***Restoration with glass-hybrid restorative system with open-sandwich technique***: After applying cavity conditioner to the exposed dentin surface for 10 s, glass-hybrid restorative system was placed along the gingival third of the proximal aspect, following the measurement of cavity depth. Then, a universal bonding agent was applied to the remaining cavity according to the manufacturer’s instructions and polymerized with an LED curing light device. Subsequently, nanohybrid composite resin was applied in 2 mm increments and polymerized with an LED curing light device.
- ***Restoration with high-viscosity glass ionomer restorative material with open-sandwich technique***: After applying cavity conditioner to the exposed dentin surface for 10 s, high-viscosity glass ionomer restorative material was placed along the gingival third of the proximal aspect following the measurement of cavity depth. Then, a universal bonding agent was applied to the remaining cavity according to the manufacturer’s instructions and polymerized with an LED curing light device. Subsequently, nanohybrid composite resin was applied in 2 mm increments and polymerized with an LED curing light device.

### Fracture resistance test

Prepared specimens were kept in distilled water at 36.5 °C in an incubator for 24 h. After 24 h, finishing and polishing procedures were performed using finishing burs and polishing disks. Subsequently, a Universal Testing Machine (Shimadzu IG-IS, Kyoto, Japan) was used for fracture resistance testing. Continuous increasing force was applied to the specimens at a cross-speed of 1 mm/min parallel to the long axis of the tooth until fracture occurred. The force at which the fracture occurred in each specimen was recorded in Newtons (N).

### Fracture analysis

The fracture surface areas of the specimens were examined under a Stereomicroscope (Olympus SZ-40, Tokyo, Japan) at x30 magnification. The fracture resistance test and the fracture analysis was performed by a different operator. Fracture analysis was performed by blind evaluation. Fracture types in the teeth were categorized into two groups: restorable and non-restorable. Restorable fractures were classified as fractures with fracture lines located no more than 1 mm below the cemento-enamel junction. Non-restorable fractures were classified as fractures with fracture lines located more than 1 mm below the cemento-enamel junction [16–18].

.

### Statistical analysis

IBM SPSS Statistics 22 software was used for statistical analysis of the obtained data in the study. The normal distribution of parameters was assessed using the Kolmogorov-Smirnov and Shapiro-Wilk tests, and it was determined that the parameters were normally distributed. Descriptive statistical methods (minimum, maximum, mean, standard deviation) were used to evaluate the study data. For comparisons of quantitative data showing normal distribution among groups, the One-Way ANOVA test was utilized, and the Tukey Honestly Significant Difference (HSD) test was used to determine the group causing the difference. For comparisons of qualitative data, Fisher’s Exact Chi-Square test and Fisher-Freeman-Halton Exact Chi-Square test were employed. The significance level was considered as *p* < 0.05.

## Results

### Fracture resistance results

There were statistically significant differences observed among the groups in terms of fracture resistance means (*p* < 0.05) (Table 2). It was observed that the fracture resistance values of Control group were significantly higher than those of GHRS, HVGIC, FB, NH, HVGIC-CS, GHRS-OS, and HVGIC-OS groups (*p* < 0.05). There was no statistically significant difference observed between the fracture resistance values of Control group and those of HVB and GHRS-CS groups (*p* > 0.05).

**Table 2:** Comparison of groups with the control group in terms of fracture resistance

	Fracture Resistance (*N*)	
	Mean±(SD)	*p*
Control group	1400.58±(395.7)	
GHRS group	992.94±(335.08)	0.023*
HVGIC group	881.74±(302.01)	0.004*
HVB group	1141.74±(372.59)	0.149
FB group	946.85±(327.77)	0.012*
NH group	872.94±(252.38)	0.002*
GHRS-CS group	1238.37±(365.78)	0.354
HVGIC-CS group	1046.79±(280.39)	0.033*
GHRS-OS group	950.78±(371.07)	0.017*
HVGIC-OS group	821.41±(305.41)	0.002*

Stdent *t* Test *p < 0.05

Abbreviations: GHRS: Glass-Hybrid Restorative System; HVGIC: High-Viscosity Glass Ionomer Restorative Material; HVB: High-Viscosity Bulk-Fill Composite Resin; FB: Flowable Bulk-Fill Composite Resin; NH: Nanohybrid Composite Resin; CS: Closed-Sandwich Technique; OS: Open-Sandwich Technique

Statistically significant differences in terms of fracture resistance were not observed between GHRS, GHRS-CS, and GHRS-OS groups (*p* > 0.05). Similarly, there was no statistically significant difference in fracture resistance between HVGIC, HVGIC-CS, and HVGIC-OS groups (*p* > 0.05). No statistically significant difference in fracture resistance was observed between GHRS-CS and GHRS-OS groups (*p* > 0.05), as well as between HVGIC-CS and HVGIC-OS groups (*p* > 0.05). The fracture resistance mean of GHRS-CS group was statistically significantly higher than that of HVGIC-OS (*p* < 0.05). There were no statistically significant differences observed in fracture resistance means among the other groups (*p* > 0.05).

### Fracture analysis results

**Fig. 3:**
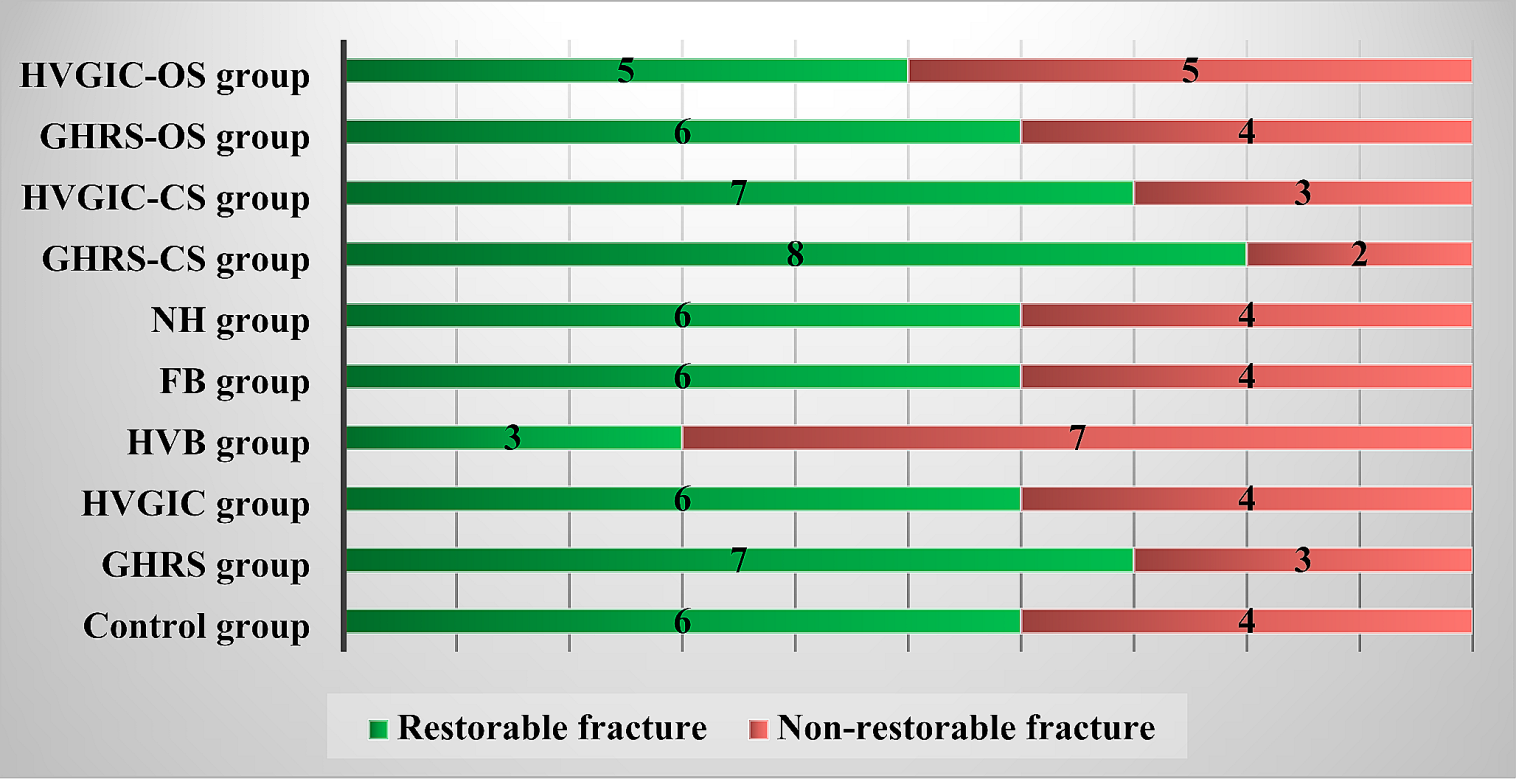
Fracture analysis values according to groups Abbreviations: GHRS: Glass-Hybrid Restorative System; HVGIC: High-Viscosity Glass Ionomer Restorative Material; HVB: High-Viscosity Bulk-Fill Composite Resin; FB: Flowable Bulk-Fill Composite Resin; NH: Nanohybrid Composite Resin; CS: Closed-Sandwich Technique; OS: Open-Sandwich Technique

Statistically significant differences were not observed among the groups in terms of fracture analysis results (*p* > 0.05) (Fig. 3).

## Discussion

The most common two reasons for the failure of composite resin restorations are bulk fracture and secondary caries. In some cases, the restoration of posterior teeth with composite resin materials can be clinically challenging. Class II restorations can lead to some restorative problems and complications; the first being adhesive failure occurring at the gingival aspect of the cavity, and the second being related to polymerization shrinkage of composite resin materials and polymerization depth. In preventing these complications, the diversity of materials and application techniques can have significant effects on the performance of restorations in the oral cavity [19]. In this *in-vitro* study, materials that can be used for the restoration of Class II cavities were evaluated, and the suitability of open and closed-sandwich techniques in terms of their effect on the fracture resistance of teeth and their restorability characteristics was assessed.

In this study, there was no statistically significant difference found between the fracture resistance values of teeth restored with high-viscosity bulk-fill composite resin and those restored using the closed-sandwich technique with glass-hybrid restorative system, compared to the fracture resistance values of intact teeth. Thus, the first and second null hypotheses of the study were rejected.

When occlusal force is applied to the tooth, the load is distributed to the bone through the periodontal ligament, thus preventing the tooth from fracturing. Periodontal ligament is a soft connective tissue that connects the tooth root to the alveolus and ensures the attachment of the tooth to the alveolar bone. It helps absorb occlusal loads and distribute them towards the bone, thus preventing teeth from fracturing [20]. There are studies in the literature that apply 90 degree load during the fracture resistance test on premolar teeth [21–26]. It is reported in the literature that when occlusal loading is applied parallel to the long axis of the teeth, the force is transmitted equally to the teeth [21]. Thus, load is applied to both cusps of the teeth at the same time [26]. Considering the previous studies, in this study, loading was done parallel to the long axis of the teeth in order to apply equal load to the cusp. Fracture resistance of teeth is directly associated with the cessation of crack propagation. Chewing forces can lead to cusp deflection. However, composite resins decrease cusp deformations under chewing load. Differences in durability among different composite resins can be attributed to variations in their matrix chemical composition, filler content, filler size, and distribution [27]. Measuring a restoration’s propensity for fracture in the oral cavity can be challenging due to many influencing factors. These factors include material composition and properties, as well as cavity size, tooth structure integrity, and bonding effectiveness [28]. Studies in the literature have reported reduced cusp deformation and higher fracture resistance with bulk-fill composite resins [29, 30]. In this study, there was no statistically significant difference found between the fracture resistance values of teeth restored with high-viscosity bulk-fill composite resin and those of intact teeth. This result may be attributed to the enhanced properties of high-viscosity bulk-fill composite resins. The high filler ratio of the high-viscosity bulk-fill composite resin used in this study, both in terms of volume and weight, may contribute to its good durability, potentially resulting in restored teeth exhibiting fracture resistance similar to intact teeth. Since fractures result from local stresses exceeding the tooth’s strength, any changes in stress concentrations due to variations in loading conditions or composite resin properties must be considered, as they could affect the tooth’s fracture resistance. The degree of conversion of a composite resin, along with its polymerization depth, also influences the development of stresses. Inadequate polymerization tends to compromise the mechanical properties of composite resin restorations, their adhesion to the tooth, and thus their long-term clinical success. Advances in initiator systems and increased translucency have been reported to result in bulk-fill composite resins exhibiting greater polymerization depth [29]. Another reason for the similar fracture resistance of teeth restored with high-viscosity bulk-fill composite resin in this study may be attributed to advancements in initiator systems and increased translucency, leading to bulk-fill composite resins showing greater polymerization depth. Tetric N-Ceram Bulk-Fill contains Ivocerin as a photoinitiator. The manufacturer asserts that Ivocerin is more reactive compared to other conventional initiators, accelerating polymerization and ensuring reliable polymerization even in deep cavities with short polymerization periods [31].

In this study, there was no statistically significant difference found between the fracture resistance values of teeth restored using the closed-sandwich technique with glass-hybrid restorative system and intact teeth. Superior outcomes regarding fracture resistance of teeth restored using the closed sandwich technique with Equia Forte may be attributed to the addition of specific components to the material. Researchers have reported that the inclusion of ultrafine and highly reactive glass particles in the material could positively influence fracture resistance values [32]. Miletić et al. evaluated the 5-year clinical performance of the glass hybrid restorative system and nano-hybrid resin composite in medium to large bifacial class II cavities. Although it was reported that the most important reason for the failure of the restorative materials evaluated in the study was fractures, as a result of the study, it was reported that Equia Forte could be one of the therapeutic options for medium to large bi-surface class II restorations of posterior teeth [33]. However, in this study, teeth where the entire cavity was restored with glass-hybrid restorative system or with nanohybrid composite resin exhibited lower fracture resistance values. In the closed-sandwich technique, glass-hybrid restorative system was placed as a base with nanohybrid composite resin applied on top. Reducing the volume of composite resin in the sandwich technique can decrease polymerization shrinkage [34]. Polymerization shrinkage and the resulting stress are important factors affecting the success of restorations made with composite resins. During the polymerization process, shrinkage occurs in both the composite resin and the tooth structure, leading to stresses. When subjected to occlusal forces, microcracks form in the tooth and at the tooth-restoration interface. These microcracks can trigger tooth fracture or marginal gap formation, ultimately resulting in restoration failure [35]. The reason for the lack of significant difference found in this study between the fracture resistance values of teeth restored using the closed-sandwich technique with glass-hybrid restorative system and intact teeth may be attributed to the reduction in polymerization shrinkage-induced stresses due to the decrease in composite resin volume, positively affecting the fracture resistance of teeth. However, in this study, the fracture resistance of teeth restored using the closed-sandwich technique with glass-hybrid restorative system was found to be lower than that of teeth restored using the closed-sandwich technique with glass-hybrid restorative system. This may be due to differences in material durability because of variations in the filler content of the glass ionomer base material used. The reinforcement mechanism of Equia Forte relies on evenly distributed ultrafine and highly reactive glass particles and optimization (increase) of the molecular weight of polyacrylic acid. This reinforcement mechanism ensures the acquisition of a material with excellent mechanical properties. The manufacturer states that this new material can be used not only for Class I and V restorations but also for Class II restorations subjected to heavy chewing loads. It is reported to have high flexural strength and resistance to wear and acid erosion. Moshaverinia et al. evaluated the physical properties of different glass ionomer restorative materials and compared Equia Forte with Fuji IX. The study reported that Equia Forte exhibited superior flexural strength and surface hardness [36]. Heck et al. evaluated the 6-year clinical performance of two different high viscosity glass ionomer cements in class II cavities. As a result of the study, both materials were reported to have acceptable and comparable survival rates after 6 years. However, researchers reported that high-viscosity glass ionomer cement may be an acceptable restoration material for smaller class II cavities. One of the materials used in the mentioned study is Equia Fil. Researchers stated that this material was further developed into a glass-hybrid material and put on the market under the name Equia Forte. In this material, more voluminous glass-filling particles are supported by smaller, highly reactive glass-filling particles. In this way, it has been reported that the material has better compressibility and less sticky consistency, thus the material can be processed better and at the same time its flexural strength increases [37]. Joshi et al. compared the compressive and flexural strengths of three different glass ionomer restorative materials. As a result of the study, it was observed that Equia Forte was the material with the highest compressive and flexural strength [38].

In this study, the fracture resistance values of teeth restored using the closed-sandwich technique with glass-hybrid restorative system were found to be higher than those of teeth restored with nanohybrid composite resin. One of the suggested methods to eliminate stresses caused by composite resin polymerization shrinkage is to use a material with excessive elastic deformation in the early stages of polymerization. Therefore, sandwich restorations, where a part of the composite resin volume is replaced with glass ionomer material, are one of the techniques recommended for improving marginal adaptation in Class I and II cavities [7]. In such restorations, the internal and external edge integrity of materials can be considered important parameters in evaluating the long-term success of the restoration. A close fit and durability between the base material and the composite resin applied to the top contribute to good stress transmission and long-term success [39]. Restoring teeth using glass-hybrid restorative system with the closed-sandwich technique may reduce the transmission of stresses from the polymerization shrinkage of nanohybrid composite resin to tooth tissues, thereby positively affecting the fracture resistance of the tooth.

In their systematic review, Parra Gatica et al. reported that flowable bulk-fill composite resins can be used in posterior region restorations in terms of monomer conversion degree, flexural strength and polymerization shrinkage, but differences between the composition of flowable bulk-fill composite resins on the market may affect the performance of the materials [40]. In general, flowable bulk-fill composite resins exhibit better performance in terms of polymerization efficiency compared to high-viscosity bulk-fill composite resins. To increase polymerization depth, attempts have been made to reduce the filler content of the material by decreasing its translucency. However, it has been noted that reduced filler content may reduce the mechanical properties of the material [41]. In this study, the fracture resistance of teeth restored with flowable bulk-fill composite resin was found to be lower than both intact teeth and teeth restored with high-viscosity bulk-fill composite resin. The reason for this may be attributed to the adverse effect on the mechanical properties of the material due to its low filler content.

The potential for fluoride release to dental tissues, along with biological and chemical compatibility, has made glass ionomer cements a special group of materials in terms of caries prevention. However, mechanical properties such as low resistance to fracture, low hardness, and wear resistance limit their use in posterior teeth subjected to heavy stress [42]. The physical properties of glass ionomer materials are influenced by factors such as powder/liquid ratio, polyacid concentration, particle size of the glass powder, and conditions during material preparation [43]. The lower filler content and presence of the liquid component in glass ionomer restorative materials compared to composite resins can lead to reduce mechanical and physical properties. Therefore, the fracture resistance of teeth restored only with high-viscosity glass ionomer restorative materials may be lower than intact teeth, teeth restored with high-viscosity bulk-fill composite resin, and teeth restored with glass-hybrid restorative system using the closed-sandwich technique.

In the open-sandwich technique, glass ionomer material forms part of the outer surface of the restoration. The proximal part of the cavity is restored with glass ionomer material, with the cement placed gingivally along the proximal to approximately the contact area or up to the occlusal wall level. The occlusal part of the cavity is then completed with composite resin [7]. Glass ionomer restorative materials are used in open-sandwich restorations to reduce microleakage and secondary caries. Although initial improvement in marginal adaptation may be observed, a high rate of clinical failure is also observed over time due to degradation in the structure of glass ionomer restorative material [44]. Küçükeşmen et al. evaluated the water sorption and solubility levels of composite, compomer, and resin-modified glass ionomer cement materials polymerized with different light sources in their study. The study reported that resin-modified glass ionomer cement exhibited the highest water sorption and solubility values [45]. Bethapudy et al. evaluated the water absorption, solubility, and microhardness of different types of glass ionomer materials in their study. The results of the study reported that Fuji IX exhibited the highest values of solubility in water. The early dissolution of glass ionomer cements in water can be attributed to two reasons: firstly, the presence of release calcium and aluminum ions that can be removed during chemical reactions of the freshly prepared material, and secondly, the formation of soluble salts in water and the presence of sodium in glass ionomer cements. Additionally, aluminum ions react slowly with the anions forming the matrix and are sensitive to early water leakage before bonding [46]. In this study, teeth were immersed in distilled water at 36.5 °C for 24 h in an incubator to simulate the oral environment before conducting the fracture resistance test. In teeth restored using the open-sandwich technique, degradation due to the solubility property of the glass ionomer restorative material exposed to distilled water could negatively affect the structure of the material and potentially reduce the fracture resistance of the teeth.

In this study, after completion of the fracture resistance test, the types of fractures in the teeth were classified into two groups: restorable and non-restorable [16–18]. However, no difference was found between the groups in the analysis conducted. The similarity of fracture types in teeth restored using different materials and techniques to intact teeth may be promising in terms of the re-restorability of teeth in clinical practice.

In this study, a standardized evaluation of the static fracture resistance of teeth restored with different materials and techniques was conducted in a laboratory setting. However, this *in-vitro* study does not completely simulate dynamic oral conditions. Various factors are more effective in the performance of restorative treatments in clinical conditions. Another limitation was the lack of periodontal ligament simulation. Therefore, the results of this study should be interpreted carefully, and further research should be conducted.

## Conclusions

Within the limitations of this study:

- The use of high-viscosity bulk-fill composite resin and the application of glass-hybrid restorative system with the closed-sandwich technique in the restoration of wide Class II cavities may be recommended due to their positive effects on the fracture resistance values of teeth.
- Clinicians should be careful about using flowable bulk-fill composite resin, nanohybrid composite resin only, and the open-sandwich technique in the restoration of teeth with such cavities, as they may reduce fracture resistance.
- Although the restorative material used may have different effects on the fracture resistance of teeth, it can be concluded that it does not lead to a significant difference in terms of the types of fractures occurring in teeth.

## Data Availability

No datasets were generated or analysed during the current study.
